# HECT E3 Ubiquitin Ligase-Regulated Txnip Degradation Facilitates TLR2-Mediated Inflammation During Group A Streptococcal Infection

**DOI:** 10.3389/fimmu.2019.02147

**Published:** 2019-09-18

**Authors:** Po-Chun Tseng, Chih-Feng Kuo, Miao-Huei Cheng, Shu-Wen Wan, Chiou-Feng Lin, Chih-Peng Chang, Yee-Shin Lin, Jiunn-Jong Wu, Chi-Chen Huang, Chia-Ling Chen

**Affiliations:** ^1^School of Respiratory Therapy, College of Medicine, Taipei Medical University, Taipei, Taiwan; ^2^Department of Microbiology and Immunology, School of Medicine, College of Medicine, Taipei Medical University, Taipei, Taiwan; ^3^School of Medicine, I-Shou University, Kaohsiung, Taiwan; ^4^Department of Nursing, I-Shou University, Kaohsiung, Taiwan; ^5^School of Medicine for International Students, College of Medicine, I-Shou University, Kaohsiung, Taiwan; ^6^Graduate Institute of Medical Sciences, Taipei Medical University, Taipei, Taiwan; ^7^Department of Microbiology and Immunology, College of Medicine, National Cheng Kung University, Tainan, Taiwan; ^8^Center of Infectious Disease and Signaling Research, National Cheng Kung University, Tainan, Taiwan; ^9^Department of Biotechnology and Laboratory Science in Medicine, School of Biomedical Science and Engineering, National Yang-Ming University, Taipei, Taiwan; ^10^Graduate Institute of Neural Regenerative Medicine, College of Medical Science and Technology, Taipei Medical University, Taipei, Taiwan; ^11^Pulmonary Research Center, Wan Fang Hospital, Taipei Medical University, Taipei, Taiwan

**Keywords:** group A *Streptococcus*, Txnip, TLR2, itch, ubiquitination

## Abstract

Thioredoxin-interacting protein (Txnip) inhibits the activity of thioredoxin (Trx) to modulate inflammatory responses. The burden of inflammation caused by microbial infection is strongly associated with disease severity; however, the role of Txnip in bacterial infection remains unclear. In Group A *Streptococcus* (GAS)-infected macrophages, Txnip was degraded independent of glucose consumption and streptococcal cysteine protease expression. Treatment with proteasome inhibitors reversed GAS-induced Txnip degradation. The activation of Toll-like receptor 2 (TLR2) initiated Txnip degradation, while no further Txnip degradation was observed in TLR2-deficient bone marrow-derived macrophages. NADPH oxidase-regulated NF-κB activation and pro-inflammatory activation were induced and accompanied by Txnip degradation during GAS infection. Silencing Txnip prompted TLR2-mediated inducible nitric oxide synthase (iNOS)/NO, TNF-α, and IL-6 production whereas the blockage of Txnip degradation by pharmacologically inhibiting the HECT E3 ubiquitin ligase with heclin and AMP-dependent protein kinase with dorsomorphin effectively reduced such effects. Our findings reveal that TLR2/NADPH oxidase-mediated Txnip proteasomal degradation facilitates pro-inflammatory cytokine production during GAS infection.

## Introduction

Recognition of Toll-like receptors (TLRs), the most important pathogen recognition receptors expressed on innate immune cells, with pathogen-associated molecular patterns can rapidly initiate the coordinated activation of transcriptional factors and result in the effective expression of pro-inflammatory mediators (1). In response to *Streptococcus pyogenes* infection, the production of pro-inflammatory cytokines is mostly regulated by TLR-myeloid differentiation factor 88 (MyD88) signaling (2, 3). Group A *Streptococcus* (GAS) infection causes various diseases ranging from mild pharyngitis and impetigo to severe necrotizing fasciitis and streptococcal toxic shock syndrome (STSS) (4). In STSS, the excessive production of various cytokines is thought to be responsible for severe systemic effects, and serum levels of TNF-α and IL-6 show the highest correlation with disease severity (5, 6).

Thioredoxin-interacting protein (Txnip), a vitamin D_3_-upregulated protein in 1α,25-dihydroxyvitamin D_3_ (1,25[OH]_2_D_3_)-treated HL-60 cells (7), acts as an endogenous inhibitor of the antioxidant thioredoxin (Trx), which is involved in a wide variety of cellular processes including the response to oxidative stress, cancer development, metabolic diseases, and inflammatory processes (8–13). The reduction of Txnip protein facilitates tumor progression, whereas the overexpression of Txnip results in the inhibition of metastasis or further triggers cells undergoing apoptosis (9, 14, 15). In addition to the pro-apoptotic role of Txnip under stress, it also plays a crucial role in the induction of reactive oxygen species (ROS)-mediated NLRP3 inflammasomes whereby initiating inflammatory responses (12, 15, 16).

As a member of the alpha-arrestin protein family, Txnip comprises a PXXP sequence for the binding of SH3 domain-containing proteins such as Trx and a PPXY sequence for the recognition of WW domain-containing proteins such as the E3 ubiquitin ligase Itch (17, 18). Itch belongs to the Nedd4-like family of E3 ubiquitin ligases and has been reported to specifically mediate the transfer of ubiquitin from E2 ubiquitin-conjugating enzymes to Txnip followed by the triggering of proteasomal degradation (18). In addition, AMP-dependent protein kinase (AMPK) has been demonstrated to phosphorylate Txnip, causing its rapid degradation during energy stress (19). Reports indicate that the TNF-α-stimulated reduction of Txnip effectively causes Trx-mediated p65 denitrosylation, which results in the increased DNA binding activities of NF-κB (20). Consistent with this, exacerbated endotoxic shock occurs along with overactivated NF-κB and excessive nitric oxide (NO) induction in Txnip-deficient mice during lipopolysaccharide (LPS) stimulation (21). Therefore, the stability of Txnip has certain pathophysiological impacts on inflammatory diseases. Txnip is a vital regulator of NF-κB activation; however, little is known about its stability in controlling inflammation during bacterial infection. In this study, we investigated TLR2/NADPH oxidase-initiated HECT E3 ubiquitin ligase-dependent Txnip degradation for cytokine induction during GAS infection.

## Materials and Methods

### Bacteria

GAS strain NZ131 (type M49) was a gift from Dr. D. R. Martin (New Zealand Communicable Disease Center, Porirua). GAS strain A20 (type M1) and *speB*-deleted SW574 were kindly provided by Dr. Y. S. Lin (National Cheng Kung University Medical College, Taiwan). A clinically isolated strain of *Staphylococcus aureus* (S2-1790) was kindly provided by Dr. C. F. Lin (Taipei Medical University, Taiwan). A fresh colony was inoculated into tryptic soy broth containing 0.5% yeast extract (TSBY) (Difco Laboratories, Detroit, MI, USA) for 16 h and then renewed with fresh TSBY broth for another 3 h incubation at 37°C. The bacterial density was determined by measuring the absorbance at 600 nm with a spectrophotometer (Beckman Instruments, Somerset, NJ, USA) and plating serial dilutions of the samples on TSBY agar for counting CFU after incubation overnight at 37°C. For the preparation of heat-killed GAS, suspended bacteria were treated at 100°C for 30 min.

### Cell Cultures and Reagents

RAW264.7 macrophage cells and THP-1 monocytic cells kindly provided by Dr. C. F. Lin (Taipei Medical University, Taiwan) were obtained from American Type Culture Collection (ATCC) and cultured in Dulbecco's modified Eagle's medium (DMEM) and RPMI 1640 (Gibco, Grand Island, NY, USA) supplemented with 10% heat-inactivated fetal bovine serum (FBS), respectively. Murine BMDMs were isolated from wild-type, *Tlr2*^−/−^, or *Nox2*^−/−^ mice, which were kindly provided by Dr. C. P. Chang and Dr. C. C. Shieh (National Cheng Kung University Medical College, Taiwan) by flushing bone marrow cells from the femurs and tibias of 6- to 10-week-old C57BL/6 mice. Animal experiments were performed according to the guidelines of the Animal Protection Act of Taiwan and the experimental protocols according to guidelines established by the Ministry of Science and Technology, Taiwan were approved by the Laboratory Animal Care and Use Committee of National Cheng Kung University. Briefly, the femurs and tibias of both legs were sterilized by 75% ethanol, and cut at the end of bone. Bone marrow cells were flushed out using syringe and maintained in RPMI (Gibco) containing 10% FBS medium. After centrifuge, bone marrow cells (1 × 10^6^) were cultured in 10 ml RPMI (Gibco) containing 10% FBS and 10 ng/ml recombinant mouse M-CSF (PeproTech, Rocky Hill, NJ, USA) for 4 days. On day 5, 5 ml of culture medium was replaced with the fresh differentiation medium (RPMI supplemented with 10% FBS and 10 ng/ml M-CSF) for additional 2 days incubation. Triplicate cultures were performed by seeding ~5 × 10^5^ cells/ml in 12-wll plates or ~2 × 10^4^ cells/ml in 96-wll plates for indicated experiments. Samples were then harvested from individual culture wells followed by the subsequent analysis. Lipoteichoic acid (LTA, catalog no. L2515) and peptidoglycans (PGNs, catalog no. 77140) from *S. aureus*, MG132, lactacystin (LAC), bafilomycin A1 (BafA1), chloroquine (CQ), N-acetylcysteine (NAC), heclin, and dorsomorphin were purchased from Sigma-Aldrich (St. Louis, MO, USA).

### Bacterial Infection

Bacteria (GAS, HK-GAS, and *S. aureus*) were prepared at the indicated multiplicity of infection (MOI) and mixed with cells in antibiotic-free culture medium followed by 1,200 rpm centrifugation for 5 min. After 1 h incubation, all culture supernatants including controls and infected groups were replaced with fresh medium containing 10 μg/ml penicillin and 50 μg/ml gentamicin for further incubation at 37°C. The time point of replacing the antibiotics containing medium is defined as zero hour post-infection. At different hours post-infection (h.p.i), cells were harvested and analyzed. In LTA or PGN treatment, cells were incubated in the culture medium containing no antibiotics and collected at indicated time points. The media glucose consumption during infection was measured using Breeze®2 blood glucose test strips and a Breeze®2 blood glucose meter (Bayer Health Care, Mishawaka, WI, USA) with a detection range of 20–600 mg/dL.

### Western Blot Analysis and Immunoprecipitation Assay

Total cell lysates were extracted using a Triton X-100-based lysis buffer (1% Triton X-100, 150 mM NaCl, 10 mM Tris, pH 7.5, 5 mM EDTA, 5 mM NaN_3_, 10 mM NaF, and 10 mM sodium pyrophosphate) with a protease inhibitor mix and phosphatase inhibitor cocktail I (Sigma) and centrifuged for 10 min at 13,300 rpm. Proteins were resolved using SDS-PAGE and then transferred to a PVDF membrane (Millipore Corporation, Billerica, MA, USA). After blocking, blots were developed with a series of antibodies against Txnip (MBL International Co, Woburn, MA, USA), Itch, ubiquitin and iNOS (Cell Signaling Technology, Beverly, MA, USA), and thioredoxin (Santa Cruz Biotechnology, Santa Cruz, CA, USA). GAPDH (Millipore Corporation) and β-actin (Santa Cruz Biotechnology) were used as internal controls. Finally, blots were hybridized with HRP-conjugated goat anti-rabbit IgG or anti-mouse IgG (Cell Signaling Technology) and developed using an ECL Western blot detection kit (Millipore Corporation) according to the manufacturer's instructions. The band intensity was measured using Image J software (NIH, Bethesda, MD, USA). For IP analysis, cell lysates were incubated with anti-Txnip Ab (5 μg) and protein G-Sepharose beads for 16 h on a roller at 4°C. The beads were isolated and washed by centrifugation followed by Western blot analysis.

### ROS Detection

ROS production was detected using a Cellular Reactive Oxygen Species Detection Assay Kit (Abcam, Cambridge, MA, USA) followed by flow cytometry (FACSCalibur; BD Biosciences, San Jose, CA, USA) analysis and fluorescence microscopic observation (Olympus BX51, Olympus, Center Valley, PA, USA). In brief, cells were infected with GAS and then coincubated with 20 μM carboxymethyl-H2-dichlorofluorescein diacetate (CM-H^2^DCFDA) fluoroprobe for 30 min at 37°C in the dark. After washing, cells were collected and analyzed using flow cytometry with the excitation at 488 nm. The emission was detected with the FL-1 channel followed by CellQuest Pro 4.0.2 software (BD Biosciences) analysis, and quantification was performed using WinMDI 2.8 software (The Scripps Institute, La Jolla, CA, USA). The percentages of ROS-positive cells each group were normalized to the mean of untreated control groups and shown.

### Cell Transfection and RNA Interference

RAW 264.7 cells were transiently transfected with catalytically inactive mouse pCINeo-myc-Itch (C832A) or siRNA oligos for Txnip by Lipofectamine reagent (Invitrogen, Carlsbad, CA, USA) according to the manufacturer's instructions. The myc-Itch (C832A) was kindly provided by Dr. L. J. Hsu (National Cheng Kung University Medical College, Taiwan). The Stealth RNAi^TM^ siRNA duplex oligoribonucleotides for Txnip (RNAi-1, 5′-UCCUCCUUGCUAUAUGGACAUCAUU-3′; RNAi-2, 5′-AAUGAUGUCCAUAUAGCAAGGAGGA-3′; RNAi-3, 5′-CCAGCCAACUCAAGAGGCAAAGAAA-3′; RNAi-4, 5′-UUUCUUUGCCUCUUGAGUUGGCUGG-3′; RNAi-5, 5′-GAGAAGAAAGUUUCCUGCAUGUUCA-3′; and RNAi-6, 5'-UGAACAUGCAGGAAACUUUCUUCUC-3′) were purchased from Invitrogen. A non-specific scrambled siRNA kit (Stealth^TM^ RNAi Negative Control Duplexes; Invitrogen) was used as the negative control. To stably express a lentivirus-based short hairpin RNA (shRNA) targeting Itch, TRCN0000026908 (5′- CCCTACGAGTAAATTATGTTT-3′) obtained from the National RNAi Core Facility (Institute of Molecular Biology/Genomic Research Center, Academia Sinica, Taiwan) was used, and TRCN0000072247 (5′-GAATCGTCGTATGCAGTGAAA-3′) was used as the control luciferase shRNA (shLuc). Lentiviral mouse Itch shRNA was obtained from the RNAi Core of Research Center of Clinical Medicine (National Cheng Kung University Hospital). Briefly, RAW 264.7 cells were infected with an appropriate MOI for 24 h followed by puromycin (Calbiochem) selection. The protein expression was then measured by Western blot analysis.

### NO and Cytokine Determination

For NO detection, nitrite (NO_2−_) accumulation in the cell culture medium was used as an indicator of NO production by the Griess reaction. Briefly, supernatants were mixed with an equal volume of Griess reagent (1% sulfanilamide, 0.1% naphthylethylenediamine dihydrochloride, and 2.5% H_3_PO_4_) and incubated for 10 min at room temperature. The relative optical density (OD) of nitrite was measured at 540 nm, and the concentration was evaluated by using sodium nitrite as a standard. TNF-α and IL-6 production were measured by enzyme-linked immunosorbent assay (ELISA) kits (R&D Systems, Minneapolis, MN, USA) according to the manufacturer's instructions.

### NF-κB Activation

RAW-Blue cells that stably express a secreted embryonic alkaline phosphatase (SEAP) reporter construct inducible by NF-κB and AP-1 were kindly provided by Dr. K. F. Hua (Department of Biotechnology and Animal Science, National Ilan University, Taiwan) and originally purchased from InvivoGen Corp. (San Diego, CA, USA). SEAP activity was measured using QUANTI-Blue SEAP detection reagent (InvivoGen Corp.) according to the manufacturer's instructions. Briefly, RAW-Blue cells were cultured in 96-well plates and infected with GAS during cotreatment with NAC. Twenty microliters of culture supernatants were collected at 24 h post-infection and then incubated with 200 μl of freshly prepared QUANTI-Blue reagent for 30 min, followed by measurement of the OD at 620–655 nm.

### Thioredoxin Activity Assay

Thioredoxin (Trx) activity was measured by using the PROTEOSTAT® Thioredoxin-1 assay kit according to manufacturer's instructions (EnZo Life Science, Plymouth Meeting, PA, USA). Briefly, cells were harvested at the indicated time points followed by total protein extraction using a Triton X-100-based lysis buffer. Samples (20 μg/10 μl) were then incubated with 70 μl of insulin containing Trx-1 Assay Master Mix and 10 μl of dithiothreitol (DTT) at room temperature for 30 min in dark. After adding 10 μl of Stop Reagent working solution, the fluorescence intensity was measured by a Varioskan Flash Multimode Reader at Ex500/Em603 nm (ThermoFisher Scientific Inc.). The fluorescence intensities of each group were then normalized to the mean of untreated control groups and shown.

### Statistics

Comparisons between two treatments were performed by unpaired *t*-test, and comparisons between various groups were performed by one-way ANOVA with GraphPad Prism version 6.0 (La Jolla, CA). Statistical significance was set at *p* < 0.05.

## Results

### GAS Infection Triggers Txnip Degradation

**Figure 1:**
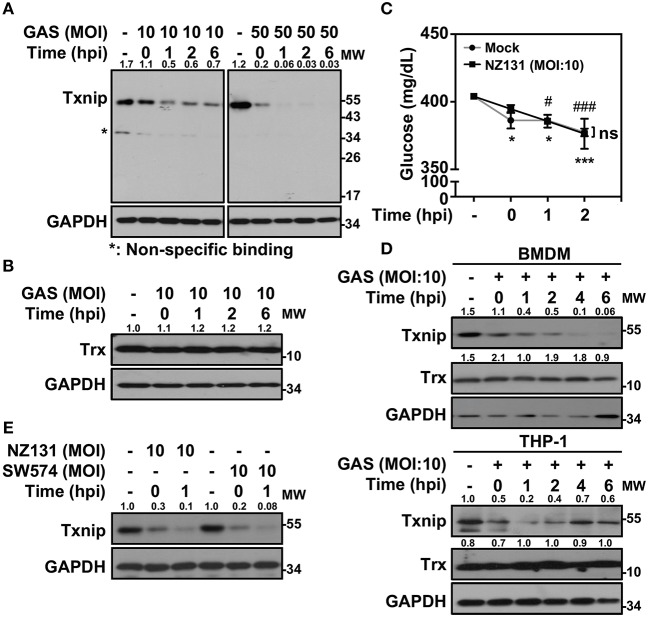
Txnip is degraded in GAS-infected macrophages. **(A)** RAW264.7 cells were infected with the indicated MOI of GAS strain NZ131 (serotype M49) for 1 h. At different hours postinfection (hpi), Txnip expression was measured using Western blot analysis. GAPDH is used as an internal control. **(B)** Expression of Trx in GAS-infected RAW 264.7 cells at the indicated hpi is shown. **(C)** The concentrations of glucose in culture media with or without GAS infection were determined at the indicated hpi. Data are shown as the means ± SD of triplicate cultures. *p < 0.05; ***p < 0.001; ^#^*p* < 0.05; ^###^*p* < 0.001 compared with the culture medium alone. **(D)** Expression of Txnip and Trx in GAS-infected BMDMs and THP-1 cells at different hpi were detected. **(E)** Txnip expression in cells infected with the *speB* wild-type strain NZ131 and the *speB*-deleted strain SW574 at the indicated hpi was measured. GAPDH is used as an internal control. The expression ratios of Txnip and Trx to internal controls are shown. Protein molecular weights (MW) are indicated in kilodaltons. Western blot results represent at least two independent experiments.

*Txnip*^−/−^ mice were extremely susceptible to LPS-induced endotoxic shock via increasing NF-κB activation and NO production (21). In Gram-positive bacterial infection, the function of Txnip in regulating inflammatory induction remains unclear. Therefore, we sought to investigate the role of Txnip in GAS-infected macrophages. Murine macrophage RAW264.7 cells were infected with different multiplicities of infection (MOIs) of GAS for 1 h, and the protein expression of Txnip was determined at different times post-infection. Txnip protein exhibited time- and dose-dependent reductions in expression during GAS infection (Figure 1A), while the expression of Trx remained similar (Figure 1B). The expression of Txnip has been shown to be tightly correlated with the extracellular concentration of glucose (22); therefore, glucose consumption during GAS infection was determined. The results indicated that glucose consumption occurred both in non-infected and GAS-infected RAW 264.7 cells along with the incubation. However, there were no significant differences of glucose consumption between non-infected and infected cells within 2 h post-infection, while Txnip had already been degraded in GAS infection (Figure 1C). Further confirmation of the changes in Txnip expression in GAS-infected bone marrow-derived macrophages (BMDMs) and naïve human monocytic THP-1 cells revealed that Txnip is susceptible to a reduction of its expression during infection (Figure 1D). Moreover, the streptococcal pyrogenic exotoxin B (SPE B) of GAS contains cysteine proteinase activity that can digest diverse host proteins including extracellular matrix proteins, immunoglobulins, complements, and opsonins (23, 24). We therefore determined the possible role of SPE B in Txnip degradation. Following the infection of the *speB* mutant strain SW574 that we generated before (25), a similar degradation of Txnip was detected following infection with the wild-type strain NZ131 and the *speB* mutant strain SW574 (Figure 1E). Txnip undergoes significant degradation independent of glucose consumption and streptococcal cysteine protease activation in GAS-infected macrophages.

### Txnip Ubiquitination and Proteasomal Degradation in GAS Infection

**Figure 2:**
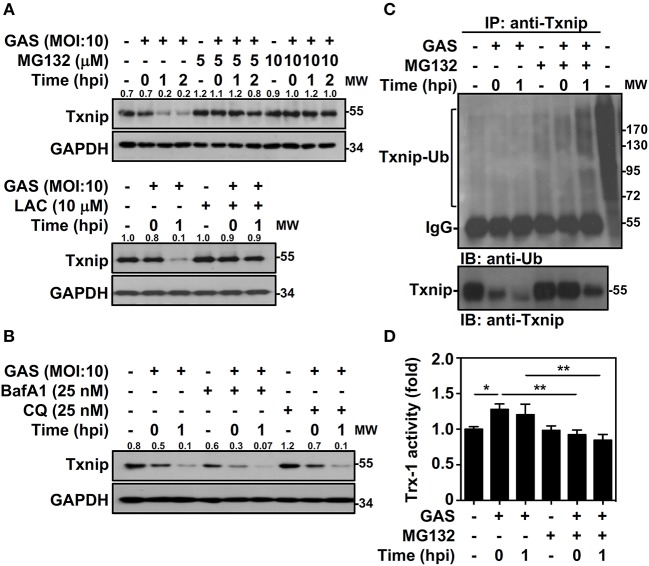
Txnip ubiquitination initiates proteasomal degradation in GAS-infected macrophages. **(A)** RAW264.7 cells were pretreated with or without MG132 and LAC for 1 h followed by GAS infection, and the expression of Txnip was determined. **(B)** In the presence or absence of BafA1 and CQ, Txnip expression in GAS-infected cells was detected. GAPDH is used as an internal control. The expression ratios of Txnip to internal controls are shown. **(C)** In the presence or absence of MG132 (10 μM), RAW264.7 cells were infected with GAS (MOI 10), and Txnip proteins were then immunoprecipitated (IP) followed by subsequent immunoblotting (IB) with anti-Ub and anti-Txnip antibodies. Cell lysate is used as the positive control and the Txnip-Ub polyubiquitination is labeled. Protein molecular weights (MW) are indicated in kilodaltons. Western blot results represent at least two independent experiments. **(D)** In the presence or absence of MG132 (10 μM), RAW264.7 cells were infected with GAS (MOI 10) followed by the measurement of Trx-1 activity at the indicated hpi. Data are shown as the means ± SD of triplicate cultures. *p < 0.05; **p < 0.01.

Txnip protein has been shown to exert relatively rapid turnover through ubiquitination-dependent degradation in human epithelial cells (18). To determine whether the decreased expression of Txnip in GAS-infected RAW264.7 cells is proteasome-mediated, proteasome-specific inhibitors were used to block proteolytic activities. The presence of MG132 and lactacystin (LAC) effectively inhibited the GAS-induced reduction in Txnip expression (Figure 2A). In addition, selective degradation mediated by autophagy was examined by using lysosomal inhibitors, and the presence of bafilomycin A1 (BafA1) and chloroquine (CQ) were unable to reverse Txnip degradation (Figure 2B). The lactate dehydrogenase (LDH) analysis showed that the concentration of MG132, LAC, BafA1, and CQ treatment showed no significant cytotoxic effects on RAW264.7 cells (Supplemental Figure 1). Although, we cannot exclude all types of cell death induced by cytotoxic agents. Since Txnip was effectively degraded by the proteasome during GAS infection, we then measured the ubiquitination of Txnip. Further immunoprecipitation analysis showed that the polyubiquitination of Txnip was increased in GAS-infected cells in a time-dependent manner in the presence of MG132 (Figure 2C). Moreover, the degradation of Txnip effectively increased the activity of Trx-1 in GAS-infected RAW264.7 cells, whereas the presence of MG132 inhibited the Trx-1 activation (Figure 2D). The protein changes of Txnip showed a correlation with Trx activation, which suggested that in GAS infection, Txnip was rapidly ubiquitinated followed by subsequent proteasomal degradation potentially leading to Trx activation.

### TLR2-dependent Txnip Degradation

**Figure 3:**
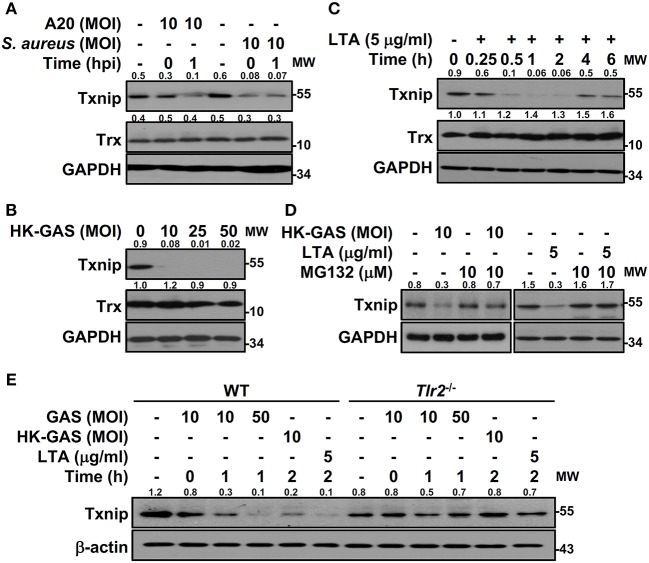
TLR2 mediates GAS-induced Txnip degradation. Western blotting showed Txnip and Trx expression in **(A)** RAW264.7 cells infected with GAS strain A20 (serotype M1) or *S. aureus*, **(B)** cells stimulated with inactivated GAS (HK-GAS) for 2 h, and **(C)** LTA-treated RAW264.7 cells. **(D)** In the presence or absence of MG132, cells were treated with HK-GAS or LTA for 2 h followed by the detection of Txnip expression. **(E)** Txnip expression was measured in wild-type (WT) or *Tlr2*^−/−^ BMDMs infected with GAS (MOI 10 and 50) or stimulated with HK-GAS or LTA for the indicated times. GAPDH and β-actin were used as internal controls. The expression ratios of Txnip and Trx to internal controls are shown. Protein molecular weights (MW) are indicated in kilodaltons. Western blot results represent at least two independent experiments.

To determine the specificity of Txnip degradation during infection, another serotype M1 GAS strain, A20, and an additional Gram-positive bacterium, *S. aureus*, were subjected to investigation. In both A20 and *S. aureus* infection, Txnip was still rapidly degraded in RAW264.7 cells (Figure 3A). Further examination of heat-killed GAS (HK-GAS) infection also showed a similar Txnip degradation pattern (Figure 3B). This implies that the recognition of TLR2 common to GAS, HK-GAS, and *S. aureus* may play a crucial role in initiating Txnip degradation. Consistent with this, the TLR2 ligand lipoteichoic acid (LTA) induced time-dependent Txnip degradation, while no obvious changes in Trx were detected (Figure 3C). MG132 treatment also blocked HK-GAS- and LTA-induced Txnip degradation in RAW264.7 cells (Figure 3D). In addition to LTA, the TLR4 ligand lipopolysaccharide (LPS) could cause Txnip degradation in BMDMs as well (Supplemental Figure 2). To further identify the involvement of TLR2 in Txnip regulation, BMDMs from *Tlr2*^−/−^ mice were infected with different MOIs of GAS or stimulated with HK-GAS and LTA. The degradation of Txnip was distinctly impeded in *Tlr2*^−/−^ BMDMs (Figure 3E). Therefore, Txnip exhibits TLR2-dependent proteasomal degradation during infection.

### NADPH Oxidase-Regulated NF-κB Activation and Txnip Degradation During GAS Infection

**Figure 4:**
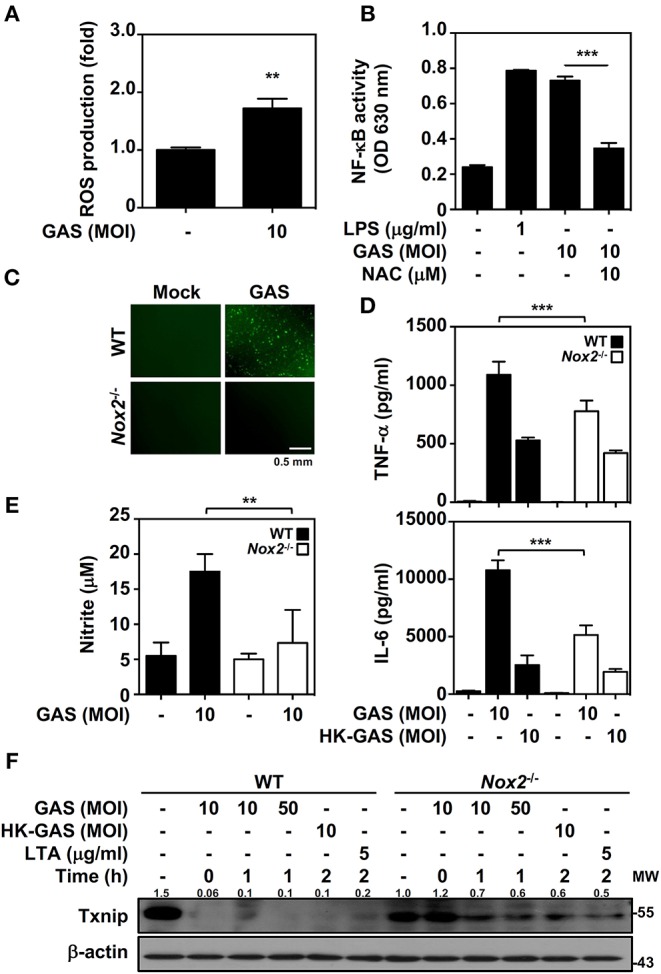
NADPH oxidase regulates NF-κB activation and Txnip degradation in GAS infection. **(A)** BMDMs were infected with GAS for 1 h followed by ROS detection. Data are shown as the means ± SD of triplicate cultures. **p < 0.01. **(B)** In the presence or absence of NAC, the relative NF-κB activities were determined in RAW-Blue cells infected with GAS for 24 h. Data are shown as the means ± SD of triplicate cultures. ***p < 0.001. LPS treatment was used as a positive control. **(C)** Fluorescence microscopic analysis showed ROS generation (*green*) in wild-type (WT) and *Nox2*^−/−^ BMDMs infected with GAS (MOI 10). The scale bar is shown. **(D)** TNF-α and IL-6 production in WT or *Nox2*^−/−^ BMDMs infected with GAS or HK-GAS for 24 h were measured and shown as the means ± SD of triplicate cultures. ***p < 0.001 compared with GAS-infected WT. **(E)** NO production in GAS-infected WT and *Nox2*^−/−^ BMDMs for 24 h was detected and shown as the means ± SD of triplicate cultures. **p < 0.01 compared with GAS-infected WT. **(F)** Western blotting showed Txnip expression in WT and *Nox2*^−/−^ BMDMs infected with GAS or treated with HK-GAS or LTA for the indicated times. β-actin was used as an internal control. The expression ratios of Txnip to internal controls are shown. Protein molecular weights (MW) are indicated in kilodaltons. Western blot results represent at least two independent experiments.

In bacterial infection, the recognition of TLR2 enhances NADPH oxidase-mediated reactive oxygen species (ROS) generation to induce bactericidal activity and inflammatory signal transduction (26, 27). We previously demonstrated that NADPH oxidase regulates GAS-initiated inflammation in macrophages (28). ROS production in GAS-infected BMDMs was therefore measured and showed a significant increase at 30 min post-infection (Figure 4A). GAS-mediated NF-κB activation could be partly blocked in the presence of the ROS scavenger N-acetylcysteine (NAC) in RAW-Blue cells (Figure 4B). BMDMs obtained from wild-type and *Nox2*^−/−^ mice were infected with GAS, and the induction of ROS was markedly inhibited in *Nox2*^−/−^ BMDMs (Figure 4C). Consistent with this, TNF-α and IL-6 production declined in *Nox2*^−/−^ BMDMs infected with GAS (Figure 4D). In addition, GAS-induced NO generation was likewise reduced in *Nox2*^−/−^ BMDMs (Figure 4E). Since NADPH oxidase is involved in TLR2-mediated signal transduction, whether TLR2-initiated Txnip degradation is susceptible to NADPH oxidase activation remains unclear. Wild-type and *Nox2*^−/−^ BMDMs were infected with GAS or stimulated with HK-GAS and LTA. The degradation of Txnip was partly inhibited in *Nox2*^−/−^ BMDMs, which also corresponded to the lower production of TNF-α, IL-6, and NO (Figure 4F). In GAS infection or TLR2 activation, Txnip exhibits NADPH oxidase-dependent degradation accompanied by NF-κB-mediated pro-inflammation.

### Txnip Degradation Prompts TLR2-mediated Inflammatory Mediator Production

**Figure 5:**
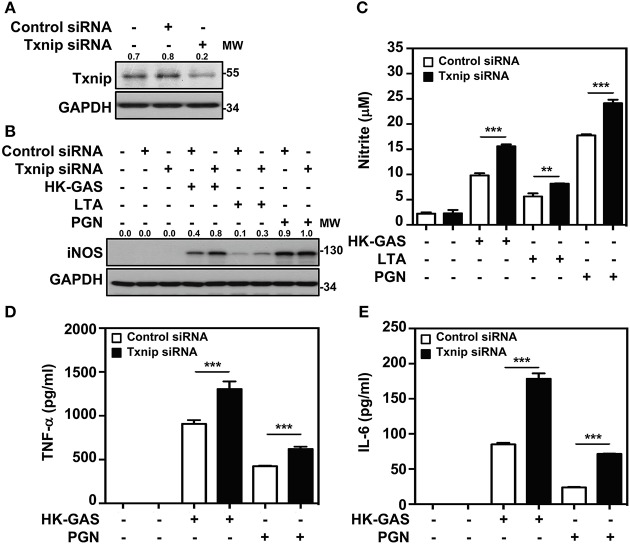
Txnip silencing facilitates TLR2-mediated iNOS/NO, TNF-α, and IL-6 production. **(A)** Western blotting showed the expression levels of Txnip and GAPDH in RAW264.7 cells transfected with control siRNA or Txnip siRNA (5 nM) for 24 h. **(B)** iNOS expression and **(C)** NO production were detected in control siRNA- or Txnip siRNA-expressing cells stimulated with HK-GAS (MOI 10), LTA (5 μg/ml), and PGN (5 μg/ml) for 24 h. GAPDH was used as an internal control. The expression ratios of Txnip and iNOS to internal controls are shown. Nitrite concentrations are shown as the means ± SD of triplicate cultures. **p < 0.01; ***p < 0.001. The levels of **(D)** TNF-α and **(E)** IL-6 were measured in control siRNA- or Txnip siRNA-expressing cells that were treated with HK-GAS or PGN for 24 h and are shown as the means ± SD of triplicate cultures. ***p < 0.001 compared with control siRNA-expressed cells. Protein molecular weights (MW) are indicated in kilodaltons. Western blot results represent at least two independent experiments.

*Txnip*^−/−^ mice were previously demonstrated to be capable of inducing abundant NF-κB activation in LPS stimulation (21). We further confirmed whether Txnip deficiency exerts similar effects on TLR2-mediated inflammatory induction. By the transient transfection of specific Txnip siRNAs into RAW264.7 cells, the expression of Txnip was suppressed (Figure 5A). HK-GAS- and LTA-mediated iNOS expression (Figure 5B), and HK-GAS-, LTA-, and PGN-initiated NO production (Figure 5C) were augmented in Txnip knockdown RAW264.7 cells. Likewise, inflammatory TNF-α and IL-6 production was profoundly enhanced in HK-GAS-, LTA-, and peptidoglycan (PGN)-stimulated Txnip knockdown cells (Figures 5D,E). Accordingly, Txnip deficiency certainly potentiates TLR2-mediated inflammatory cytokine induction.

### Itch-independent Txnip Degradation in Response to TLR2 Signals

**Figure 6:**
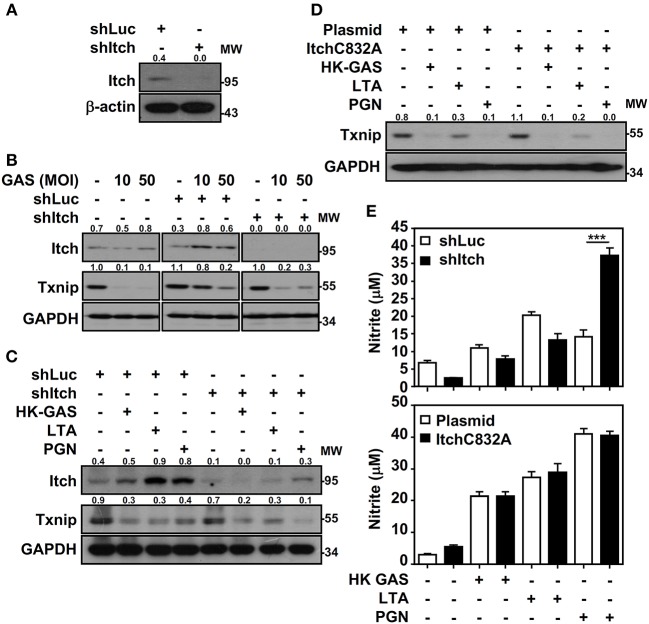
Itch-independent Txnip degradation promotes TLR2-mediated NO production. **(A)** RAW264.7 cells stably expressed control luciferase shRNA (shLuc) and Itch shRNA (shItch). The expression of Itch and β-actin was measured. Western blotting showed Itch and Txnip expression in WT, shLuc-, and shItch-expressed RAW264.7 cells **(B)** infected with the indicated MOIs of GAS at 2 h postinfection or **(C)** stimulated with HK-GAS (MOI 10), LTA (5 μg/ml), and PGN (5 μg/ml) for 2 h. GAPDH was used as an internal control. **(D)** Txnip expression was detected in cells transiently transfected with a dominant-negative mutant of Itch (C832A) followed by HK-GAS, LTA, and PGN treatment for 2 h. GAPDH is shown as an internal control. The expression ratios of Txnip and Itch to internal controls are shown. **(E)** shLuc- or shItch-expressing (*upper panel*) and control plasmid- or ItchC832A-expressing (*lower panel*) RAW264.7 cells were treated with HK-GAS, LTA, and PGN for 24 h. NO production was measured and shown as the means ± SD of triplicate cultures. ***p < 0.001 compared with shLuc-expressed cells. Protein molecular weights (MW) are indicated in kilodaltons. Western blot results represent at least two independent experiments.

Itch E3 ubiquitin protein ligase has been demonstrated to directly interact with Txnip and act as a robust ubiquitin ligase for Txnip (18); the interaction between Txnip and Itch during GAS infection is therefore verified. Immunoprecipitation analysis revealed that there were very minimum amounts of Txnip interacting with Itch in GAS-infected RAW264.7 cells (data not shown). To further examine Itch-mediated Txnip degradation, Itch knockdown RAW264.7 cells were used (Figure 6A). Interestingly, Txnip protein remained degraded either in GAS-infected wild-type and control shLuc-cells or in GAS-infected shItch-cells (Figure 6B). Further stimulation of HK-GAS, LTA and PGN also revealed the similar degradation of Txnip in both control shLuc- and shItch-cells (Figure 6C). Itch is similar to other members of the E3 ligase family that utilize the critical cysteine residue in the HECT domain to mediate substrate ubiquitination (29); the mutation of this cysteine in Itch to alanine (C832A) was applied accordingly to validate Txnip degradation in response to TLR2 signals. Similarly, the overexpression of catalytically inactive Itch (C832A mutation) in RAW264.7 cells did not efficiently inhibit GAS-induced Txnip degradation (Figure 6D). Meanwhile, NO production remained similar in wild-type, Itch knockdown or Itch-mutated cells following HK-GAS, LTA, and PGN stimulation (Figure 6E). Moreover, JNK-regulated phosphorylation and activation of Itch is reported to initiate c-FLIP proteasomal degradation in TNF-α stimulation (30). In the presence of JNK and p38 inhibitor, Txnip remained being degraded during GAS infection (Supplemental Figure 3). Accordingly, we speculate that TLR2 activation could rapidly initiate the ubiquitination and proteasomal degradation of Txnip independent of Itch activation.

### AMPK- and HECT E3 Ubiquitin Ligase-Regulated Txnip Degradation in TLR2 Activation

**Figure 7:**
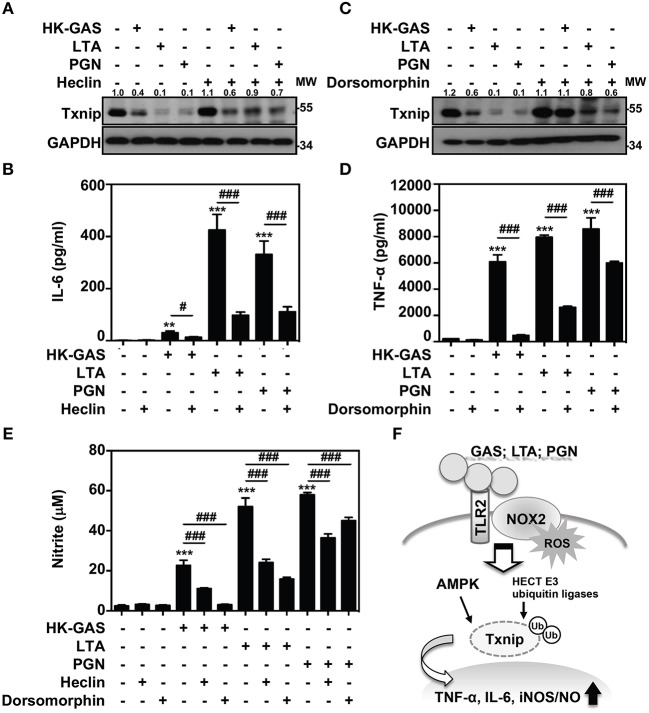
AMPK and HECT E3 ligase are required for Txnip degradation in TLR2-mediated inflammation. RAW264.7 cells were pretreated with **(A,B)** heclin (25 μM) or **(C,D)** dorsomorphin (10 μM) for 1 h followed by stimulation with HK-GAS (MOI 10), LTA (5 μg/ml), and PGN (5 μg/ml). The expression of Txnip and GAPDH was detected at 2 h by Western blotting. The expression ratios of Txnip to GAPDH are shown. Protein molecular weights (MW) are indicated in kilodaltons. Western blot results represent at least two independent experiments. ELISA showed IL-6 and TNF-α production at 24 h in HK-GAS-, LTA-, and PGN-stimulated cells, and the concentrations were shown as the means ± SD of triplicate cultures. **p < 0.01 and ***p < 0.001 compared to the untreated group; ^#^*p* < 0.05 and ^###^*p* < 0.001 compared to each stimulated group. **(E)** In the presence of heclin and dorsomorphin, the Griess reaction showed NO generation in HK-GAS-, LTA-, and PGN-stimulated cells at 24 h. The measurements are shown as the means ± SD of triplicate cultures. ***p < 0.001 compared to the untreated group; ^###^*p* < 0.001 compared to each stimulated group. **(F)** A represented model of that GAS infection induces the TLR2/NOX2-dependent rapid degradation of Txnip via AMPK- and HECT E3-ligase-regulation, which subsequently potentiates downstream inflammatory TNF-α, IL-6, and iNOS/NO generation.

Itch belongs to the Nedd4-like family of E3 ubiquitin ligases, which also contains additional members including Nedd4, Nedd4-2, Smurf1, WWP1, WWP2, NEDL1, and NEDL2 (31, 32). Because the blockage of Itch was insufficient to cease TLR2-mediated Txnip degradation, the involvement of other HECT E3 ubiquitin ligases (HECT E3s) was then investigated. A small molecular inhibitor, heclin, has been identified that specifically causes a conformational change of the HECT domain in Nedd4, Smurf2 and WWP1, resulting in the oxidation of the active site cysteine and inhibiting ligase activity (33). We found that the presence of heclin suppressed TLR2-induced Txnip degradation in RAW264.7 cells (Figure 7A). Similarly, HK-GAS-, LTA-, and PGN-mediated Txnip degradation could be partly reversed by heclin treatment in primary murine peritoneal macrophages as well (Supplemental Figure 4A). Consistent with the stabilization of Txnip, TLR2-activated IL-6 production in RAW264.7 cells was significantly reduced in the presence of heclin (Figure 7B). Nedd4, Smurf2, or WWP1 could be another essential E3 ubiquitin ligase that contributes to Txnip proteasomal degradation in TLR2 activation.

In addition, AMPK has been reported to phosphorylate Txnip, initiating its rapid degradation during energy stress (19). The phosphorylation of AMPK at threonine 172 (Thr172) in TLR2 activation was then measured. Results showed that HK-GAS, LTA, and PGN stimulation induced the rapid AMPK phosphorylation at Thr172 in RAW264.7 cells (Supplemental Figure 5). Interestingly, the inhibition of AMPK by using its pharmaceutical inhibitor dorsomorphin in RAW264.7 cells distinctly obstructed TLR2-mediated Txnip degradation (Figure 7C) as well as TNF-α production (Figure 7D). TLR2-mediated Txnip degradation were likewise reversed by dorsomorphin treatment in murine peritoneal macrophages (Supplemental Figure 4B). Dorsomorphin is also used as an inhibitor for the bone morphogenetic protein (BMP) signaling (34). In HK-GAS-, LTA-, and PGN-stimulated RAW264.7 cells, the phosphorylation of BMP-mediated Smad1/5/8 was unaffected (Supplemental Figure 6A). Dorsomorphin treatment impeded TLR2-mediated Txnip degradation dose-dependently in RAW264.7 cells, while the phosphorylation of Smad1/5/8 remained unaffected (Supplemental Figure 6B). It suggests that AMPK activation regulates TLR2-mediated Txnip degradation. The present concentration of heclin and dorsomorphin treatment showed no significant cytotoxic effects on RAW264.7 cells (Supplemental Figure 1). Moreover, TLR2-activated NO production was significantly inhibited in the presence of heclin and dorsomorphin along with stable levels of Txnip (Figure 7E). Therefore, the decreased production of IL-6, TNF-α, and NO is consistent with the stabilization of Txnip, which indicates that TLR2-mediated inflammation is partly regulated by Txnip expression.

## Discussion

In GAS infection, macrophages play a crucial role in the host innate defense against bacteria and in pro-inflammatory induction. The recognition of GAS with pattern recognition receptors (PRRs) such as TLRs can effectively trigger signaling cascades that elicit NF-κB activation and inflammatory cytokine induction (35). The engagement of TLRs launches MyD88-dependent functional inflammatory responses, while MyD88 deficiency results in the distinct reduction in the levels of TNF-α, IL-6, IL-12 and interferons in GAS-infected phagocytes (2, 35). PRRs certainly mediate the fundamental immune responses for defense against bacterial infection; however, GAS-derived TLR activation and subsequent inflammatory mechanisms are not completely understood. Here, we first demonstrate that GAS initiated the rapid proteasomal degradation of Txnip, which is independent of glucose consumption or SPE B activation. GAS-mediated Txnip degradation exhibited TLR2- and NOX2-dependent regulation, which were also expressed in concert with the production of inflammatory cytokines. Moreover, the blockage of HECT E3s and AMPK could stabilize Txnip expression whereby partly reducing TNF-α, IL-6, and NO generation in TLR2-activated macrophages. Accordingly, a represented model is provided and indicated that in response to GAS infection or LTA and PGN stimulation, the activation of TLR2 could sufficiently coordinate NOX2-producing ROS to potentiate AMPK- and HECT E3s-regulated Txnip degradation, resulting in inflammatory TNF-α, IL-6, and iNOS/NO production (Figure 7F).

As a stress-active protein, Txnip is involved in a wide variety of cellular processes. Txnip effectively interacts with Trx, resulting in the decreased ability of Trx to reduce downstream substrates (36). Trx acts as an oxidoreductase and denitrosylase to regulate NF-κB activity through direct interaction with the p50/p65 heterodimer, reducing the redox-sensitive cysteine in the Rel domain and enhancing the ability of NF-κB to bind DNA (37, 38). Txnip degradation has been demonstrated to contribute to Trx activation, which in turn facilities TNF-α-stimulated NF-κB activation in the respiratory epithelium (20). In addition, *Txnip*^−/−^ mice display a higher susceptibility to NF-κB induction during TNF-α stimulation, which subsequently promotes diethylnitrosamine-induced hepatocarcinogenesis (39). Txnip deficiency aggravates LPS-induced endotoxic shock and *E. coli* infection-induced mortality through excessive NO production (21). However, in *P. aeruginosa*-induced bacteremic shock, Txnip inhibits macrophage phagocytosis by phosphatidylinositide 3-kinase inactivation and ROS degeneration, resulting in the inhibition of bacterial clearance (40). In GAS infection, Txnip degradation is involved in TLR2-mediated inflammatory induction, in which the suppression of Txnip causes magnified TNF-α, IL-6, and NO production. The potential role of Txnip in streptococcal toxic shock can be anticipated.

Txnip was previously shown to be rapidly degraded during cytokine and LPS stimulation, while the mechanisms regulating its proteasomal degradation remain unknown. Txnip normally forms a stable complex with Itch and maintains a relatively rapid turnover in 293T and U2OS cells. The WW domain of Itch interacts with the PPXY region of Txnip, and the highly conserved cysteine residue in the HECT domain of Itch triggers the ubiquitination of Txnip (18). In GAS-infected macrophages, we found that Txnip could be polyubiquitinated and partially form a complex with Itch. However, both the specific silencing of Itch and the overexpression of inactive Itch ligase mutants (Itch-C832A) were insufficient to impede TLR2-initiated Txnip degradation, which suggests an additional E3 ubiquitin ligase that might manipulate TLR2-mediated Txnip degradation. Heclin is a broad inhibitor of a range of HECT E3s, particularly for the specific inhibition of Nedd4, Smurf2, WWP1, WWP2, and Nedd4L (33). The presence of heclin partly stabilized the expression of Txnip, while TLR2-activated inflammatory cytokines were reduced. This finding indicates that HECT E3s play a crucial role in TLR2-initiated Txnip degradation and inflammation. In the present study, we confirmed an Itch-independent Txnip degradation in TLR2 and GAS stimulation; however, the specific HECT E3s in labeling Txnip for proteasomal degradation remains unclear. Therefore, further deciphering the detailed mechanisms of TLR2-mediated Txnip degradation by using specific siRNAs will be an urgent issue.

During GAS infection, M protein, the pore-forming toxin streptolysin O, and NAD-glycohydrolase have been shown to regulate the NLRP3 inflammasome, resulting in the maturation and release of the pro-inflammatory cytokine IL-1β (41–43). Inflammasome induction represents an early warning sign in bacterial infection; however, hyperinflammation often aggravates tissue injury by causing systemic shock. Txnip has been revealed to dissociate from oxidized Trx that initiates the NLRP3 inflammasome in response to ROS activation (12, 36). So far, the role of Txnip in bacteria-incited inflammasomes has not been reported. In LPS or *E. coli* infection, Txnip-deficient macrophages manifest the partial decrease of active caspase-1 and IL-1β production, which might result from increased S-nitrosylation of NLRP3 inflammasome components that hinder IL-1β maturation (21). Because the TLR4-regulated inflammasome shows incomplete obstruction with Txnip deficiency, the Txnip/NLRP3-independent inflammasome may present in TLR signals. While TLR2 and GAS infection mediate the rapid degradation of Txnip, it increases exacerbated NO and inflammation by intensifying NF-κB activation. Therefore, Txnip participates in GAS-mediated inflammasome induction which remains to be further investigated.

GAS infection initiates the assembly of NOX2 in macrophages, which often induces abundant ROS generation (28). NOX2-derived ROS not only exert bacterial killing but also transduce inflammatory signals and protein synthesis (28, 44). We previously demonstrate that GAS infection initiates NOX2-regulated glycogen synthase kinase-3β activation whereby promoting the activation of NF-κB in RAW264.7 cells (28, 45). In calcium oxalate crystal stimulation, NOX-mediated ROS are the crucial contributors that trigger Txnip dissociation from Trx and binding to NLRP3, which causes renal injury and inflammation (46). Here, we found that GAS increased ROS-associated Txnip degradation to potentially expedite NF-κB activation, whereas NOX2 deficiency stabilized Txnip and reduced the generation of inflammatory cytokines. Moreover, *Nox2*^−/−^ BMDMs presented partial Txnip degradation during GAS infection or HK-GAS and LTA stimulation, while almost no obvious degradation occurred in *Tlr2*^−/−^ cells, suggesting that the stability of Txnip might be regulated through the TLR2/NOX2 axis. In addition to the ROS-regulated phosphorylation of IκBα and MAPK phosphatases (47, 48), we speculate that Txnip may serve as an additional downstream responder following TLR2/NOX2 signaling to potentiate NF-κB-mediated inflammation. In GAS-infected epithelial cells, the enriched transcription factor networks, including activator protein-1, activating transcription factor 2, and nuclear factor of activated T cells, are reported to play the crucial role in induction of proinflammation (49). Besides NF-κB, the involvement of Txnip degradation in regulating other transcription factors during GAS infection is worthwhile to study further. Moreover, the redox- and energy-responsive AMPK (50, 51) shows the part regulation on TLR2/NOX2-mediated Txnip degradation, whether ROS generation potentially initiates AMPK activation which needs to be further investigated.

In contrast to the anticipated role of Txnip in the induction of inflammasomes, GAS infection triggers the rapid degradation of Txnip that results primarily in abundant TNF-α, IL-6, and iNOS/NO production. Because the excessive generation of inflammatory cytokines is frequently associated with the severity of infectious disease, we speculate that the stabilization of Txnip could be a potential target to decelerate inflammation. Interestingly, two blockers, heclin and dorsomorphin, which are specialized to inhibit HECT E3s and AMPK, respectively, can prevent Txnip degradation and subsequent inflammatory cytokine production. Their application in the treatment of hyperinflammation and detailed mechanisms of their involvement in Txnip regulation during bacterial infection require further investigation.

## Ethics Statement

The animal experiments were performed according to the guidelines of the Animal Protection Act of Taiwan and the experimental protocols according to guidelines established by the Ministry of Science and Technology, Taiwan were approved by the Laboratory Animal Care and Use Committee of National Cheng Kung University.

## Author Contributions

P-CT, C-FK, C-FL, Y-SL, and C-LC participated in the study design and data interpretation and drafted the manuscript. P-CT and C-LC performed the experiments. C-FK, M-HC, S-WW, C-PC, and J-JW assisted with experimental design and material preparation. C-LC wrote the first draft of the manuscript. C-FL, Y-SL, J-JW, and C-CH contributed to manuscript revision. All authors read and approved the final manuscript.

### Conflict of Interest Statement

The authors declare that the research was conducted in the absence of any commercial or financial relationships that could be construed as a potential conflict of interest.
