# Supplementary material for: HECT E3 Ubiquitin Ligase-Regulated Txnip Degradation Facilitates TLR2-Mediated Inflammation During Group A Streptococcal Infection
Source: Front Immunol. 2019 Sep 18;10:2147. doi: 10.3389/fimmu.2019.02147 (PMC6759821; doi:10.3389/fimmu.2019.02147)
Supplement: Supplementary file 1 [file Data_Sheet_1.PDF]

Supplementary Material

Supplementary Figures

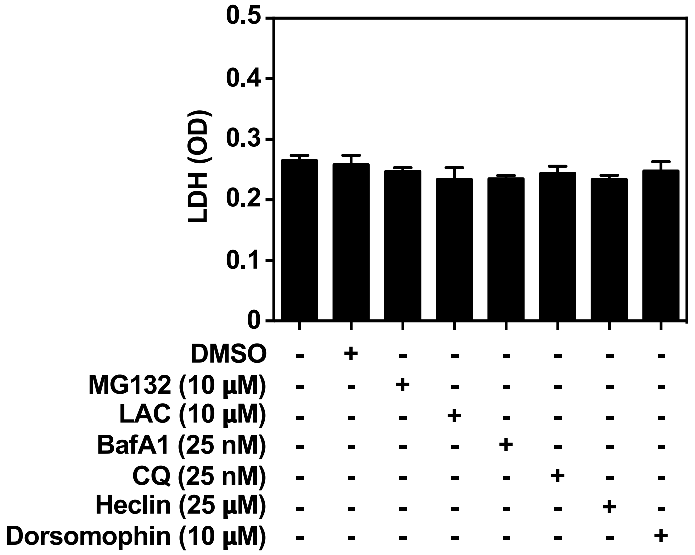

Supplementary Figure 1. *Cytotoxic effects of inhibitors on RAW264.7 cells.* RAW264.7 cells were treated with or without indicated concentration of inhibitors for 3 h. The culture supernatants were collected and measured the releasing of lactate dehydrogenase (LDH) by using Cytotoxicity Detection kit assays (Roche Diagnostics, Lewes, UK). Data are shown as the means ± SD of triplicate cultures.

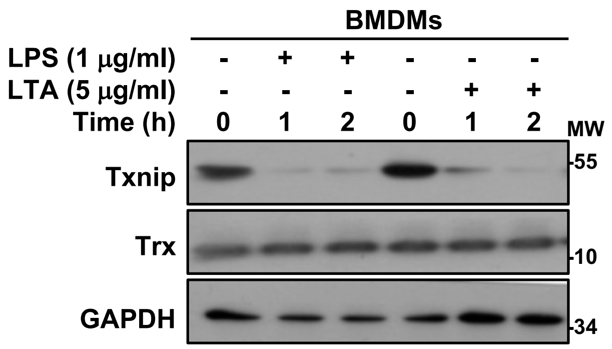

Supplementary Figure 2. *Lipopolysaccharide (LPS) and LTA induce Txnip degradation in bone marrow-derived macrophages (BMDMs).* BMDM cells were stimulated with LPS from *E. coli* (Sigma-Aldrich, catalog no. L2630) and LTA from *S. aureus* (Sigma-Aldrich, catalog no. L2515) for the indicated times followed by the Western blotting analysis for Txnip and Trx expression. GAPDH is used as the internal control and protein molecular weights (MW) are indicated in kilodaltons.

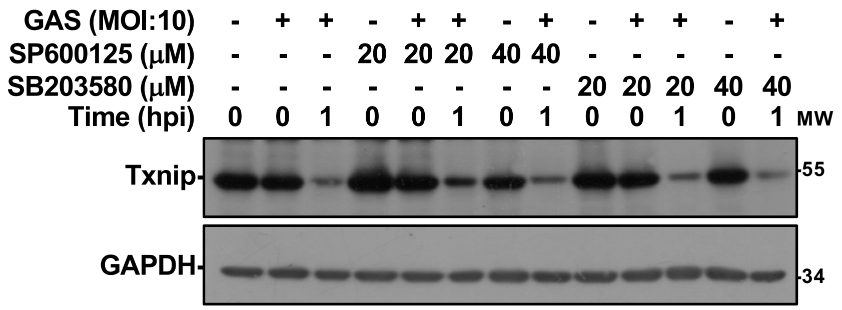

Supplementary Figure 3. *JNK- and p38-independent Txnip degradation in GAS-infected RAW264.7 cells*. In the presence of JNK inhibitor, SP600125 (TOCRIS, CAS Number: 129-56-6), and p38 inhibitor, SB203580 (TOCRIS, CAS Number: 152121-47-6), cells were infected with GAS followed by the Western blotting analysis for Txnip expression. GAPDH is used as the internal control and protein molecular weights (MW) are indicated in kilodaltons.

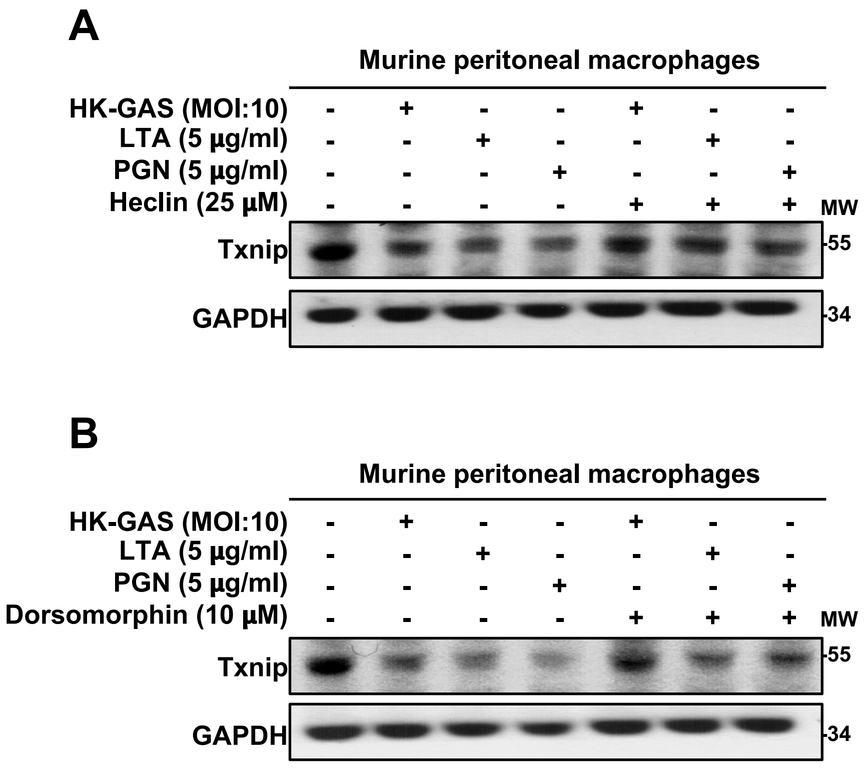

Supplementary Figure 4. *Heclin and dorsomorphin inhibit HK-GAS-, LTA-, and PGN-mediated Txnip degradation in murine peritoneal macrophages.* The resident peritoneal macrophages were isolated from 6~8 week-old C57BL/6 mice by injection of RPMI medium (5 ml) and aspiration of fluid from peritoneum. After washing and attaching, cells were pretreated with heclin **(A)** and dorsormophin **(B)** for 1 h followed by HK-GAS, LTA, and PGN stimulation for 2 h. The expression of Txnip was measured by Western blotting analysis. GAPDH is used as the internal control and protein molecular weights (MW) are indicated in kilodaltons.

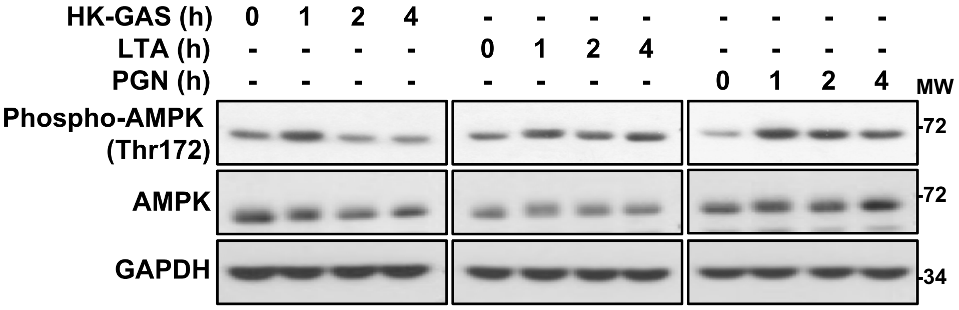

Supplementary Figure 5. *HK-GAS, LTA, and PGN induce AMPK phosphorylation in RAW264.7 cells.* Cells were treated with HK-GAS (MOI: 10), LTA (5 μg/ml), and PGN (5 μg/ml) for the indicated times. The phosphorylation of AMPK at threonine 172 (Thr172) and the expression of AMPK were detected using specific antibodies against phospho-AMPK and AMPK (Cell Signaling Technology, Beverly, MA, USA). GAPDH is used as the internal control and protein molecular weights (MW) are indicated in kilodaltons.

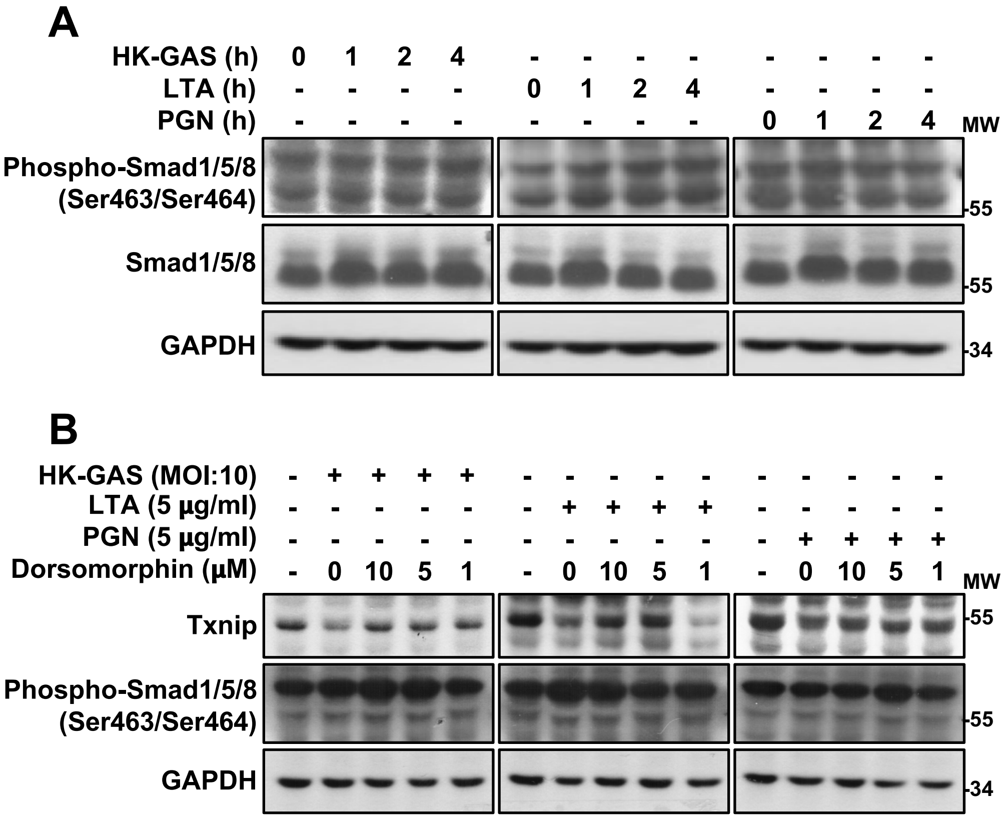

Supplementary Figure 6. *Dorsomorphin inhibits TLR2-mediated Txnip degradation dose-dependently in RAW264.7 cells.* **(A)** Cells were treated with HK-GAS (MOI: 10), LTA (5 μg/ml), and PGN (5 μg/ml) for the indicated times. The phosphorylation of bone morphogenetic protein (BMP)-regulated Smad1/5/8 at serine 463/serine 464 and Smad1/5/8 expression were measured using specific antibodies (Santa Cruz Biotechnology, Santa Cruz, CA, USA). **(B)** With or without indicated concentrations of dorsomorphin, HK-GAS-, LTA-, and PGN-treated cells were harvested and measured the expression of Txnip and phospho-Smad1/5/8 (Ser463/Ser464) by Western blotting analysis. GAPDH is used as the internal control and protein molecular weights (MW) are indicated in kilodaltons.
